# Effective components of integrated motivational interviewing and cognitive behavioural therapy for lifestyle behaviour change: a modified Delphi study

**DOI:** 10.1186/s12966-025-01816-6

**Published:** 2025-11-03

**Authors:** Stephen Barrett, Stephen Begg, Ashley R. Dunford, Paul O’Halloran, Jeff Breckon, Emily Denniss, Kane Rodda, Sarah Hardcastle, Paul W. Marshall, Tim Anstiss, Dominika Kwasnicka, Benjamin Bohman, Cathy Atkinson, Nicholas F. Taylor, Cameron Randall, Colin Greaves, Kate Hall, Brea Kunstler, Jeroen Lakerveld, Denise Goodwin, Cheryce Harrison, Roland Devlieger, Dane Halliwell, Daniel Faustino-Silva, Michel Kingsley

**Affiliations:** 1 Research and Innovation, Bendigo Health, Barnard St, Victoria, VIC 3552 Australia; 2 Holsworth Research Initiative, La Trobe University, Bendigo, VIC 3552 Australia; 3 La Trobe Rural Health School, Bendigo, VIC Australia; 4 Renal Services, Bendigo Health, Victoria, 3552 Australia; 5 Centre for Sport and Social Impact, La Trobe University, Melbourne, 3086 Australia; 6 School of Psychology and Public Health, La Trobe University Melbourne, 3086 Melbourne, Australia; 7 School of Health & Life Sciences, Teesside University, Middlesbrough, TS1 3BA North Yorkshire UK; 8 Institute for Physical Activity and Nutrition, School of Health and Social Development, Deakin University, Geelong, Australia; 9 Outpatient Rehabilitation Services, Bendigo Health, Victoria, 3552 Australia; 10 School of Sport and Physical Activity, Sheffield Hallam University, Sheffield, UK; 11 Department of Exercise Sciences, University of Auckland, Auckland, New Zealand; 12 Henley Business School, University of Reading, Reading, UK; 13 Centre for Health Equity, School of Population and Global Health, University of Melbourne, Melbourne, Australia; 14 Department of Clinical Neuroscience, Centre for Psychiatry Research, Karolinska Institutet & Stockholm Health Care Services, Region Stockholm, Stockholm, Sweden; 15 University of Manchester, Manchester, UK; 16 Allied Health Clinical Research Office, Eastern Health, Box Hill, VIC 3086, 3128 Australia; 17 School of Allied Health, Human Services and Sport, La Trobe University, Bundoora, VIC Australia; 18 Department of Oral Health Sciences, University of Washington School of Dentistry, Seattle, WA USA; 19 School of Sport, Exercise & Rehabilitation Sciences, University of Birmingham, Birmingham, United Kingdom; 20 School of Psychology, Deakin University, Burwood, VIC Australia; 21 Monash Sustainable Development Institute, BehaviourWorks Australia, Monash University, Victoria, 3800 Australia; 22 Department of Epidemiology and Data Science, Amsterdam UMC, Vrije Universiteit Amsterdam, De Boelelaan 1117, 1081HV, Amsterdam, the Netherlands; 23 Monash Centre for Health Research & Implementation, Monash University, Melbourne, Australia; 24 Department of Obstetrics and Gynaecology, University Hospital Leuven, Leuven, Belgium; 25 Department of Development and Regeneration, REALIFE Research group, KULeuven, Belgium; 26 Grupo Hospitalar Conceição (GHC), Brazil, Brazil; 27 Department of Exercise Sciences, University of Auckland, Auckland, New Zealand

**Keywords:** Health promotion, Behaviour change, Physical activity, Dietary changes, Smoking cessation

## Abstract

**Background:**

There are high levels of ill health across the world, largely due to lifestyle risk factors such as inadequate physical activity, unhealthy diet, and smoking. Behaviour change interventions are widely recommended for addressing many of these risk factors. While integrated motivational interviewing and cognitive behavioural therapy (MI-CBT) is increasingly used to support behaviour change, there is currently no established consensus on its core effective components. The aim of this study was to establish expert consensus on the essential elements of MI-CBT interventions for lifestyle behaviour change.

**Methods:**

A modified Delphi study comprised of three iterative online surveys involving international experts in MI-CBT and lifestyle behaviour change was conducted. Using key literature and practice guides, a list of 63 commonly used components in individually delivered motivational interviewing (MI) and cognitive behavioural therapy (CBT) interventions were developed. In each round, experts rated their agreement with each component using a Likert scale. Components reaching at least 80% agreement in Rounds 2 or 3 were included in the final list of essential MI-CBT components.

**Results:**

Thirty experts with a median of 13 years of experience in behaviour change intervention design and delivery participated in Round 1, with 28 (93%) completing Round 2, and 25 (83%) completing Round 3. Consensus was achieved for 26 components. Of these, eight were relational components which included open-ended questions, affirmations, reflections, summaries, emphasising autonomy and offering emotional support. Sixteen components were based on cognitive behavioural content, and included exploring change expectations, identifying and exploring avoidant behaviour, identifying past successes, and technical strategies such as activity scheduling and relapse prevention. Finally, two process components emphasised the importance of scheduling sessions flexibly based on client needs, and ensuring that the therapist meets recognised standards for MI-CBT training and practice.

**Conclusions:**

Expert consensus was established regarding the essential elements of MI-CBT interventions for lifestyle behaviour change. This consensus provides guidance on essential elements to include in integrated MI-CBT interventions, which may strengthen MI-CBT training, improve intervention fidelity, and enhance real-world and research applications targeting physical activity, dietary intake, and smoking cessation. The proposed consensus framework offers a foundation for future trials assessing the effectiveness and implementation of integrated MI-CBT interventions.

**Supplementary Information:**

The online version contains supplementary material available at 10.1186/s12966-025-01816-6.

## Introduction

Chronic diseases such as diabetes, cardiovascular disease, and certain cancers remain leading contributors to global morbidity and mortality [1, 2]. Risk of chronic disease is largely driven by modifiable behaviours, including low levels of physical activity (PA), poor diet quality, and smoking [3]. Addressing these modifiable behavioural risk factors through targeted interventions is essential for attenuating preventable disease burden and slowing disease progression [4]. Consequently, behaviour change interventions are widely used as a primary strategy for addressing and managing these risks [5]. A variety of theoretical approaches have been used to facilitate behaviour change, with motivational interviewing (MI) and cognitive behaviour therapy (CBT) being two of the most widely applied. MI and CBT have been increasingly integrated within behaviour change interventions to enhance their effectiveness in supporting sustained behaviour change [6–8].

MI is a well-established, evidence-based approach that seeks to facilitate behaviour change by enhancing an individual’s intrinsic motivation and resolving ambivalence toward change [9]. MI has demonstrated efficacy in initiating health behaviour change [10, 11] and emerging evidence suggests that MI may have a role in relapse prevention following lifestyle behaviour change [7]. However, many MI interventions lack explicit maintenance strategies, which may contribute to relapse [9]. Research suggests that MI is more effective and produces more sustained behaviour change when integrated with complementary therapeutic approaches, such as CBT, rather than being delivered as a standalone intervention [12]. CBT, with its emphasis on structured goal- and action-oriented strategies, and cognitive restructuring techniques, is commonly used to support both the initiation and maintenance of behaviour change [13]. While MI and CBT are increasingly integrated within behaviour change interventions [6, 8, 14–16], their distinct therapeutic styles present challenges that should be considered when designing effective interventions [17, 18].

MI is characterised by a client-centred, empathetic communication style that aligns with the “MI spirit,” encompassing partnership, acceptance, evocation, and compassion [9]. The approach is structured around four interrelated tasks: engaging (developing a collaborative relationship), focusing (clarifying and defining the target behaviour), evoking (drawing out the client’s own reasons and motivations for change), and planning (committing to actionable steps for change) [9]. The technical execution of MI relies on the proficiency of the practitioner in core micro-skills, including open-ended questions, affirmations, reflective listening, and summarising (OARS), to evoke and reinforce change talk while reducing sustain talk [9].

Similarly, CBT consists of core therapeutic components, with emphasis varying according to client needs [19]. A fundamental aspect of CBT is its focus on identifying and modifying maladaptive thoughts and cognitive patterns that influence behaviour. By exploring and restructuring these cognitions, individuals can develop more adaptive beliefs that support sustained behaviour change [13]. Although different CBT components may hold varying levels of importance for different behaviours, certain foundational elements such as goal-setting, problem-solving, and skills training are relevant across multiple behaviours. However, there is limited evidence identifying which CBT components are most essential when integrated with MI for changes in PA, diet, and smoking [20].

Two systematic reviews, both limited to randomised controlled trials, examined the effectiveness of integrated MI-CBT interventions for PA and anthropometric outcomes [6, 8]. One review focused specifically on pregnant women [6], while the other reviewed trials were conducted in a range of populations, including but not limited to, chronic fatigue, physically inactive cancer survivors, and individuals with chronic conditions such as rheumatoid arthritis or cardiovascular disease [8]. The results of these reviews highlight inconsistencies in the description and reporting of integrated MI-CBT components, as well as a lack of detail regarding intervention delivery [6, 8]. Many studies included in those reviews failed to measure intervention fidelity, despite the availability of both MI and integrated MI-CBT fidelity scales [21, 22]; this raises concerns about whether the integrated MI-CBT interventions were delivered as intended. Without clear guidance on the essential components of integrated MI-CBT interventions, consistency remains a challenge, limiting both research reproducibility and practical application. While MI and CBT are designed to be adaptable and client-centred, this flexibility can contribute to wide variation in how interventions are implemented. Identifying core components is not intended to constrain practitioner responsiveness, but rather to support shared understanding, improve fidelity, and enhance the consistency of reporting, particularly in applied health settings where MI-CBT interventions are delivered by diverse practitioners [8]. Establishing expert consensus on these components can help improve intervention clarity, replicability, and effectiveness.

A Delphi approach provides a structured, systematic method to address this gap by determining expert agreement on essential intervention components [23]. Delphi approaches typically involve multiple rounds of surveys completed by a panel of experts, with anonymised group feedback provided between rounds to refine and converge opinions [23, 24]. Consensus is generally defined as a predetermined level of agreement across the panel (e.g., ≥ 80% agreement) [23, 24]. This approach has been used to identify core elements of CBT for psychosis [25] and depression [26], but has not yet been applied to defining the essential components of integrated MI-CBT for lifestyle behaviour change.

The aim of this study was to establish expert consensus on the essential components of integrated MI-CBT interventions for lifestyle behaviour change using a modified Delphi approach. The findings will inform the design, training, and implementation of integrated MI-CBT interventions, contributing to the development of more effective and standardised behaviour change strategies.

## Methods

### Study design

A modified Delphi method was conducted following the guidance on Conducting and REporting of DElphi Studies (CREDES) checklist (Additional file 1) [27]. It is advised that the number of Delphi survey rounds be established a priori, with three rounds typically regarded as optimal [28]. Consequently, three survey rounds were pre-specified. Data collection occurred between May 2024 and August 2024. Survey data were collected using the REDCap platform (Vanderbilt University, TN). The protocol for this study has been detailed previously [29].

### Consensus

There is a lack of guidelines and agreement in the literature as to what constitutes consensus for Delphi studies. Nevertheless, most modified Delphi studies designed to establish agreed definitions in health science research have defined consensus as ≥ 70%, ≥ 75%, or ≥ 80% agreement [23, 24]. For this study, consensus was defined a priori as ≥ 80% agreement either for inclusion (‘consensus in’) or exclusion (‘consensus out’) of a component; this threshold was chosen as the most stringent level of agreement that has been previously adopted [23, 24]. It was also decided a priori that all components would be carried forward from Round 1 to Round 2 regardless of the level of agreement [29]. Only in Rounds 2 and 3 was it possible for components to reach ‘consensus in’ or ‘consensus out’ [29].

### Steering committee

A Steering Committee consisting of the study authors (SB, AD, SB, PO’H, ED, KR, JB and MK) guided the research. The committee members were clinicians and academics and were located in Australia, New Zealand and the United Kingdom.

### Participants and recruitment

A literature search was carried out using the PubMed database to identify an international panel of academics with expertise in MI-CBT for lifestyle behaviour changes [30]. A list of authors (first, senior) who had published at least one paper on the topic of MI-CBT for lifestyle behaviour changes, and whose email addresses were publicly available was created. In addition, a search was carried out to identify first and senior authors of books, book chapters, practice guidelines and grey literature on MI-CBT for lifestyle behaviour changes.

Potential participants included academics, researchers, and published authors in the MI-CBT field, as well as academics, researchers, and healthcare professionals involved in the design, implementation, or delivery of integrated MI-CBT interventions. Consistent with published Delphi studies, participants required a minimum of three years of direct experience in integrated MI-CBT to be eligible [31]. While there are no universally accepted guidelines for expert panel size in modified Delphi studies, a minimum of 20 participants has been recommended [24]. To account for attrition, which is common in Delphi studies due to their iterative nature, 47 potential experts were identified and received a personalised email invitation to participate and a link to the first survey. A snowball sampling approach was also employed, requesting recipients to forward the study invitation to others with relevant expertise, particularly clinicians whose names may not appear in manuscript or book searches. During open recruitment, the research lead (SB) advertised open invitations to potential experts using social media platforms X (formerly Twitter) and LinkedIn. Members of the research team (SB, PO’H, JB, MK) also provided study information to potential experts they knew through professional networks.

### Survey development

The MI-CBT experts on the research team compiled a comprehensive list of MI-CBT components, categorising them into three key domains: (1) MI relational and technical components (e.g., OARS), which encompass listening techniques and communication styles (the “MI spirit”); (2) content components, which include technical strategies aimed at facilitating behaviour change, such as problem-solving and activity scheduling; and (3) process components, which relate to the structure and delivery of the intervention, including the provider, session frequency, and mode of delivery. The initial list was informed by established taxonomies and fidelity tools used in MI and CBT literature, including existing research identifying relational, content-based, and process-oriented techniques within MI and CBT when delivered independently [26, 32–34]. A ‘long-list’ of 72 components was initially established. This list was iteratively refined by members of the research team (SB, SB, AD, PO’H, JB, MK) to ensure clarity and reduce redundancy, with 60 components included for assessment in Round 1. The included components and definitions are provided in additional file 2.

### Data collection

#### Round 1 survey

**Fig. 1:**
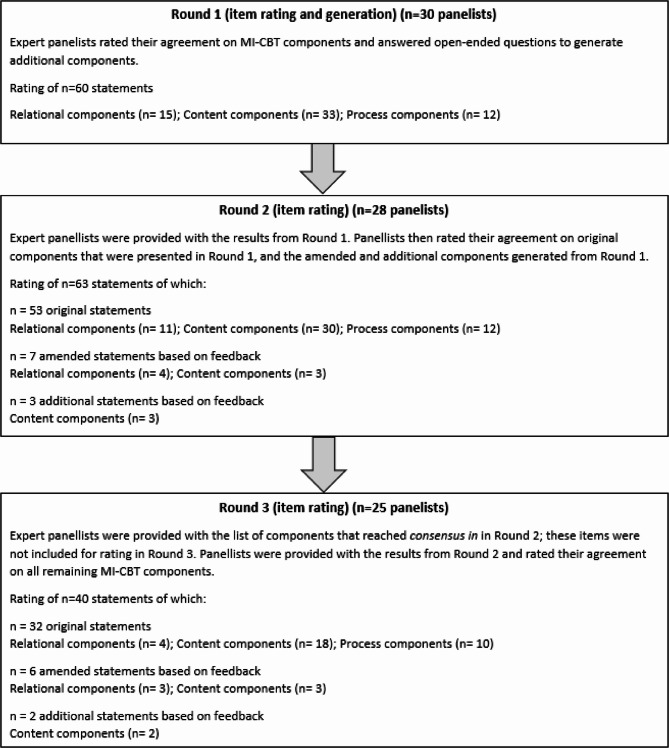
Flow diagram presenting the modified Delphi study process

The flow of the study process is presented in Fig. 1. In section one, participants were asked to provide demographic information, including their role, area of expertise, years of experience in the field, and country of employment. In section two, participants were asked to identify if they were rating MI-CBT components from the perspective of either behaviour uptake interventions (e.g. promoting PA) or behaviour cessation interventions (e.g. stopping smoking). Each participant rated all components from their selective perspective only. This approach allowed us to examine whether differences emerged depending on which perspective was adopted.

**Fig. 2:**
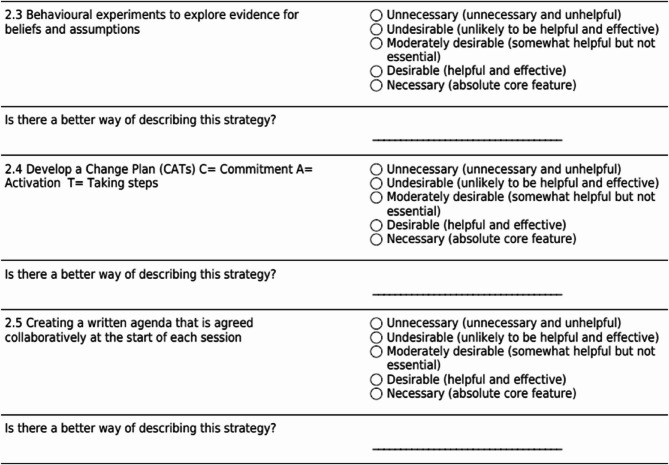
Example of statements and answers and the free text option provided to participants

Participants rated each MI-CBT component on a 5-point Likert scale, with the following wording used: ‘Please select whether you think the following components are either: 1. Necessary (absolute core features); 2. Desirable (helpful and effective); 3. Moderately desirable (somewhat helpful but not essential); 4. Undesirable (unlikely to be helpful and effective); or 5. Unnecessary (unnecessary*)’*. A 5-point Likert scale is regarded as the most effective format for evaluating components within Delphi research [28]. For each component, participants were provided with the opportunity to offer a better description if they considered it appropriate. Participants were provided with open-ended options to suggest other components they might consider necessary or desirable for all three categories (relational, content, process). Figure 2 shows a screen shot of one of the survey statements and answers, and the free text option provided to participants.

Free-text responses provided in the Round 1 survey were reviewed by the research team and discussed in relation to the overall survey responses. Recommendations that were made by several participants were given greater weight in the discussions when considering the proposed changes and finalising items for Round 2.

## Round 2

In Round 2, participants were shown the groups’ responses to every component from Round 1. Responses were presented in two bar graphs for each component: one graph summarised the responses across each category of the 5-point Likert scale and provided the count of responses for each, while the other aggregated responses into three categories: ‘necessary and desirable,’ ‘moderately desirable,’ and ‘unnecessary and undesirable'. This was the only deviation from the published protocol, where we indicated that ‘responses from Round 1 will be presented in a bar graph and include the median and IQR of participants’ responses. The research team determined that two bar graphs more completely conveyed the range of responses, rendering median and IQR statistics unnecessary.

Round 2 also incorporated modifications based on Round 1 feedback, with revised wording and definitions highlighted for participants. Participants were asked to rate all the MI-CBT components on a 5-point Likert scale, including those that achieved > 80% consensus in Round 1. It was decided a priori that all components would be re-evaluated in Round 2, regardless of their prior consensus status, as free-text feedback may lead to refinements; this process is consistent with modified Delphi approaches [30]. Participants were encouraged to consider the groups’ responses to the Round 1 survey before finalising rating [35]. No additional free-text questions were included in Round 2.

### Round 3

For Round 3, participants were again presented with bar graph results from Round 2 and asked to consider the group’s responses before finalising their opinion on the components. Components that achieved *consensus in* or *consensus out* in Round 2 were not included in Round 3.

### Data analysis

Descriptive statistics (proportions) were used to summarise participants’ demographic characteristics and their responses to each survey component. Responses to ‘necessary’ and ‘desirable’ were combined to create a ‘required’ category, while responses to ‘unnecessary’ and ‘undesirable’ were combined to create an ‘unrequired’ category. Components that reached the predetermined consensus threshold of ≥ 80% as ‘required’ were considered *consensus in*; components that reached the predetermined consensus threshold of ≤ 80% as ‘unrequired’ were considered *consensus out* [36].

### Results

In total, 30 experts participated in Round 1. Of these participants, 28 (93%) completed Round 2, and 25 (83%) completed Round 3. The median MI-CBT experience among experts was 13 years (IQR: 6 to 20). There was an almost equal representation of female (*n* = 16; 53%) and male (*n* = 14; 47%) experts. Among participants, 27% (*n* = 8) were research-focused academics, 13% (*n* = 4) combined research and teaching, 17% (*n* = 5) were clinicians, 23% (*n* = 7) were clinician-researchers, and 20% (*n* = 6) combined clinical practice, research, and teaching responsibilities. The experts came from nine different countries, with the largest groups from Australia (*n* = 9), the United States (*n* = 6), and the United Kingdom (*n* = 6). Other countries included Brazil, Canada, and the Netherlands (*n* = 2 each), and Belgium, New Zealand, and Sweden (*n* = 1 each).

#### Round 1

Sixty components were evaluated in Round 1. Of these, 21 attained ≥ 80% agreement as necessary and desirable (Table 1). As specified a priori, all components from Round 1 were carried forward to Round 2, including those that attained ≥ 80% agreement. Based on participants' feedback, nine of the 60 original components were reworded for Round 2, and definitions for components were added or amended. In addition, based on the open text responses, three new components were added to Round 2 (Fig. 2). As a result, 63 components were included in Round 2.

**Table 1:**
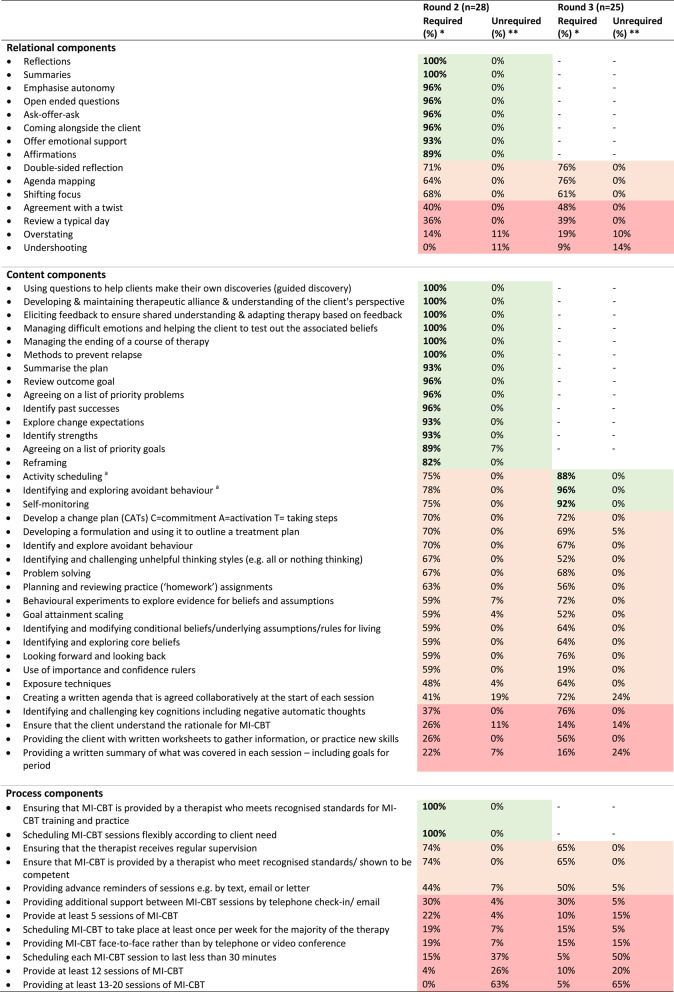
Scoring for all components in Round 2 and 3

Components in bold and green indicate reaching ≥80% agreement and classified as *consensus in*

Components in amber indicate reaching ≥40% and ≤79% agreement

Components in red indicate reaching ≥0% and ≤39% agreement

*Abbreviations*: *MI-CBT* motivational interviewing and cognitive behavioural therapy

^a^Modified statement in Round 2 based on participant feedback

*Percentage of participants reporting ‘necessary’ or ‘desirable’

**Percentage of participants reporting ‘unnecessary’ or ‘undesirable’

#### Round 2

Participants evaluated 63 components in Round 2. Of these, 23 components reached the predefined consensus threshold of ≥ 80% agreement and were therefore classified as *consensus in*. These included 8 relational components, 13 content components and 2 process components (Table 1). Descriptive statistics for all Round 2 and 3 responses not achieving consensus are included in Table 1. The components that met the *consensus in* threshold were not included in Round 3. As a result, 40 components were included in Round 3.

#### Round 3

In Round 3, 40 components were evaluated. Of these, 3 components reached the predefined consensus threshold of ≥ 80% agreement and were therefore classified as *consensus in* (Table 1). As a result, the total number of components that reached *consensus in* was 26 (8 relational, 16 content, and 2 process components). No components reached the predefined consensus threshold of ≥ 80% agreement as unnecessary and undesirable, and therefore no components were classified as *consensus out*.

#### Stratified by uptake or cessation

In Table 2, the 26 components that attained ≥ 80% agreement for *consensus in* are presented, stratified by whether experts evaluated MI-CBT components from the perspective of behaviour uptake or behaviour cessation interventions. The level of agreement with the response options of the 5-point Likert scale (necessary, desirable, moderately desirable, undesirable, unnecessary) are also presented. This details where experts considered the components to be necessary or desirable.

**Table 2:** MI-CBT components achieving consensus-in stratified by uptake and cessation behaviours

**Component**		**Combined Behaviours**^a^ **(n=28)**	**Uptake behaviours**	**Cessation behaviours**
			**(n=23)**	**(n=5)**
**Open ended questions**	Necessary (absolute core feature)	27	22	5
	Desirable (helpful and effective)	1	1	0
**Affirmations**	Necessary (absolute core feature)	9	6	3
	Desirable (helpful and effective)	16	14	2
	Moderately desirable	3	3	0
**Reflections**	Necessary (absolute core feature)	21	16	5
	Desirable (helpful and effective)	7	7	0
**Summaries**	Necessary (absolute core feature)	14	11	3
	Desirable (helpful and effective)	14	12	2
**Emphasise autonomy**	Necessary (absolute core feature)	24	19	5
	Desirable (helpful and effective)	3	3	0
	Moderately desirable	1	1	0
**Coming alongside the client**	Necessary (absolute core feature)	17	14	3
	Desirable (helpful and effective)	10	8	2
	Moderately desirable	1	1	0
**Offer emotional support**	Necessary (absolute core feature)	13	9	4
	Desirable (helpful and effective)	13	12	1
	Moderately desirable	2	2	0
**Ask-offer-ask**	Necessary (absolute core feature)	18	15	3
	Desirable (helpful and effective)	9	7	2
	Moderately desirable	1	1	0
**Agreeing on a list of priority problems**	Necessary (absolute core feature)	15	13	2
	Desirable (helpful and effective)	12	9	3
	Moderately desirable	1	1	0
**Developing and maintaining a good therapeutic alliance and understanding of the client's perspective**	Necessary (absolute core feature)	26	22	4
	Desirable (helpful and effective)	2	1	1
**Eliciting feedback to ensure a shared understanding and adapting therapy based on feedback**	Necessary (absolute core feature)	16	12	4
	Desirable (helpful and effective)	12	11	1
**Agreeing on a list of priority goals**	Necessary (absolute core feature)	10	9	1
	Desirable (helpful and effective)	15	11	4
	Moderately desirable	1	1	0
	Undesirable	2	2	0
**Explore change expectations**	Necessary (absolute core feature)	12	9	3
	Desirable (helpful and effective)	14	12	2
	Moderately desirable	2	2	0
**Identify strengths**	Necessary (absolute core feature)	13	9	4
	Desirable (helpful and effective)	13	12	1
	Moderately desirable	2	2	0
**Identify past successes**	Necessary (absolute core feature)	9	5	4
	Desirable (helpful and effective)	18	17	1
	Moderately desirable	1	1	0
**Reframing**	Necessary (absolute core feature)	9	8	1
	Desirable (helpful and effective)	14	12	2
	Moderately desirable	5	3	2
**Methods to prevent relapse**	Necessary (absolute core feature)	24	20	4
	Desirable (helpful and effective)	4	3	1
**Managing difficult emotions and helping the client to test out the associated beliefs**	Necessary (absolute core feature)	9	7	2
	Desirable (helpful and effective)	19	16	3
**Managing the ending of a course of therapy**	Necessary (absolute core feature)	17	14	3
	Desirable (helpful and effective)	11	9	2
**Summarise the plan**	Necessary (absolute core feature)	17	14	3
	Desirable (helpful and effective)	9	8	1
	Moderately desirable	2	1	1
**Review outcome goal**	Necessary (absolute core feature)	9	8	1
	Desirable (helpful and effective)	18	15	3
	Moderately desirable	1	0	1
**Ensuring that MI-CBT is provided by a therapist who meets recognised standards for MI-CBT training and practice**	Necessary (absolute core feature)	9	7	2
	Desirable (helpful and effective)	19	16	3
**Scheduling MI-CBT sessions flexibly according to client need**	Necessary (absolute core feature)	10	6	4
	Desirable (helpful and effective)	18	17	1

		**(n=25)**^b^	**(n=19)**^b^	**(n=6)**^b^
**Identifying and exploring avoidant behaviour**^b^	Necessary (absolute core feature)	10	8	2
	Desirable (helpful and effective)	14	11	3
	Moderately desirable	1	0	1
**Self-monitoring**^b^	Necessary (absolute core feature)	13	11	2
	Desirable (helpful and effective)	10	7	3
	Moderately desirable	2	1	1
**Activity scheduling**^b^	Necessary (absolute core feature)	5	5	0
	Desirable (helpful and effective)	17	12	5
	Moderately desirable	3	2	1

*Abbreviations*: *MI-CBT*Motivational interviewing and cognitive behaviour therapy

^a^Combined Behaviours **= **pooled results from uptake behaviour and cessation behaviour combined

^b^These components were deemed *consensus in* after Round 3 which had a n=25

Of the 26 components, three components, ‘reframing,’ ‘summarise the plan,’ and ‘review outcome goal', did not reach ≥ 80% agreement *consensus in* for cessation behaviours but did for client uptake behaviours. The remaining 23 components achieved ≥ 80% agreement, and were classified as *consensus in* for both uptake and cessation interventions.

### Discussion

To the best of our knowledge, this is the first systematic attempt to gain expert opinion on the most essential components of integrated MI-CBT interventions for lifestyle behaviour change (e.g. PA, diet and smoking). After employing a modified Delphi approach, 26 components across relational, content, and process categories reached *consensus in*, creating an agreed-upon list of core elements deemed essential for integrated MI-CBT interventions. The findings contribute to a clearer understanding of the components that experts deem essential for MI-CBT interventions, with important implications for future training, clinical practice, and research.

Eight relational components were identified as essential for MI-CBT interventions targeting lifestyle behaviour change; the majority of these components align with core elements of MI. Fundamental MI skills of open-ended questions, affirmations, reflective listening and summaries (OARS) help build rapport, encourage exploration and guide individuals toward self-determined motivation for change [9]. A fundamental principle of MI is emphasising autonomy, recognising individuals’ capacity to make independent decisions regarding behaviour change [9]. Rather than a collection of technical components, MI is characterised by a distinct “spirit,” conceptualised as a collaborative way of being with people [37]. Person-centred communication is pivotal in behaviour change interventions [38]; the relational components identified in this consensus statement reinforce that change occurs within individuals and through meaningful interactions as partners, rather than through professionals’ intent alone [37].

Of the sixteen content components, many reflect general therapeutic components, for example developing therapeutic alliance and eliciting feedback (typical within MI and CBT), as opposed to items specific to CBT interventions alone. Other content components might be considered as behavioural techniques (activity scheduling; self-monitoring) or cognitive techniques (reframing, exploring change expectations and identifying past successes). Many Beckian CBT concepts such as identification and challenging of key cognitions and behavioural experiments to explore evidence for beliefs and assumptions may be useful techniques to employ in some cases [13, 39], but they did not reach *consensus in*. This is similar to findings from an expert consensus on effective components for CBT for depression [26]. The content components that reached *consensus in* closely align with well-established behaviour change techniques [40], for example goal setting (behaviour), problem solving, self-monitoring of behaviour and identification of self as role model. This aligns with broader psychotherapy literature which highlights that developing a strong therapeutic relationship, setting clear expectations, and facilitating action-orientated techniques that promote health are fundamental factors that drive behaviour change across therapeutic approaches [41]. The consensus reached in this study reflects these principles, reinforcing that effective MI-CBT interventions are not defined solely by their behavioural techniques but also by the relational and process-driven elements that underpin engagement and sustained change [41].

Integrating MI and CBT components leverages the complementary strengths of both approaches, addressing different stages and aspects of behaviour change. Both MI and CBT interventions emphasise collaboration; however, they can differ in how they position the role of the therapist. CBT traditionally emphasises an expert model, where the therapist is assumed to be the key driver of change [37]. In MI, the client is explicitly seen as the expert, and the key driver of change. Rather than viewing these approaches as separate, MI can enhance CBT by aligning behaviour change with an individual’s values, strengthening intrinsic motivation [17, 18]. While values exploration is not always a central focus in health behaviour change interventions, it is a key feature of MI that may strengthen CBT’s impact when integrated [32]. Miller [42] suggested that CBT can be directed towards change based on the client’s core values, rather than adhering to a fixed view of which beliefs are considered ‘rational’ or ‘irrational’. The bidirectional influence between MI and CBT emphasises the potential for an integrated approach to improve both motivation and skill development, ensuring interventions are both client-centred and action-oriented [9, 17, 18, 37].

Several authors have suggested that relational components of interventions interact with technical or content components to influence behaviour change [43, 44]. Hilton and Johnston argued that *how* interventions are delivered is often as important as the specific content of the intervention [45]. Intervention design for behaviour change may focus too heavily on the technical components of specific therapy models, while underestimating the impact that therapist relational skill may have on outcomes [37]. Rather than viewing relational and content-based components as separate influences, their interaction likely determines effectiveness [32]. Studies demonstrate that therapeutic relational factors have a strong impact on outcomes, potentially moderating the effectiveness of specific behaviour change techniques [46]. Hardcastle and colleagues contend that content-based techniques are likely to be more effective when delivered within an interpersonal style that supports autonomy and engagement [43]. This reinforces the importance of not only identifying key intervention components but also ensuring they are embedded within a therapeutic approach that promotes motivation and self-efficacy.

While 26 components reached consensus, several did not. Non-consensus components such as ‘agenda mapping’ and ‘double-sided reflection’ may still offer clinical utility depending on the context, phase of intervention, and client needs. Their exclusion does not indicate ineffectiveness, rather, it suggests these techniques may be more phase- or condition-specific. A small number of content-based techniques also narrowly missed the consensus threshold (76%), including ‘looking forward/looking back’ and ‘identifying and challenging key cognitions’. Divergence in views on these components may reflect differences in theoretical orientation among panellists. For instance, those with a stronger CBT background may view challenging cognitions as essential to behaviour change [47], while MI specialists may consider it less relevant or potentially unhelpful depending on client readiness [48]. Flexibility and individualisation are central to both MI and CBT, and the value of non-consensus components may emerge in specific clinical contexts. However, such flexibility presents challenges for research and training. MI-CBT interventions are often poorly reported, with limited detail on what components were used, how they were delivered, and how fidelity was assessed [6, 8]. This lack of standardisation impacts replication and limits the ability to understand what makes interventions effective. By identifying components with broad expert agreement, this study provides a foundational reference to support clearer reporting, enhance fidelity monitoring, and guide training. The consensus components should therefore not be viewed as a fixed protocol, but as a practical framework to support transparency and rigour in MI-CBT intervention design, while still allowing for therapeutic responsiveness and adaptability.

Three components (reframing, summarise the plan, and review outcome goal) reached consensus for client uptake but not cessation behaviours. One potential explanation is the difference in panel size: the cessation panel was much smaller (*n* = 5), which may have limited the likelihood of reaching the 80% agreement threshold due to the greater influence of individual ratings. The small group size limits definitive interpretation. Future research with a larger panel focused on cessation behaviours could help clarify these findings.

#### Strengths and limitations

This study has several strengths. It included experts from a variety of global regions, though despite the authors’ efforts to recruit from diverse areas, there was no representation from Asia and Africa. This may reflect challenges in engaging experts from these regions, rather than a limitation of the study itself. The participants comprised a mix of research and teaching academics and clinicians with a broad range of experience, ensuring a well-rounded perspective. Additionally, expert participation exceeded the recommended minimum sample size of 20 participants for all three rounds of this modified Delphi, strengthening the reliability of the findings.

Nevertheless, there are some limitations to consider. The Delphi technique aims to recruit a targeted expert subset of the population rather than a representative sample which might limit the generalisability of the findings to the broader population of CBT experts. As a definitive list of MI-CBT experts was not available, participant selection relied on investigator-compiled lists gained from first and senior author and snowball sampling, which may have introduced selection bias. Snowball sampling, a common approach in Delphi studies, may have led to a group with shared perspectives, potentially influencing the consensus process.

Another key limitation is that the survey did not differentiate between phases of intervention. Components may be differentially important depending on the phase of intervention, for example MI is often used in early stages to enhance motivation, whereas action-oriented processes are more dominant when motivation is stable, focusing on behaviour change strategies. This phase-dependent distinction may have influenced the perceived effectiveness and importance of specific MI and CBT components. Relatedly, some techniques narrowly missed our 80% consensus threshold, such as agenda mapping and double-sided reflection, both of which achieved over 75% agreement, may have been viewed as more or less relevant depending on the stage of intervention or the health condition in question. For instance, double-sided reflection may be particularly useful when clients are ambivalent about change, while agenda mapping could be especially relevant when working with clients managing multiple risk factors (e.g., someone with hypertension, elevated cholesterol, and low PA) where collaboratively deciding which issue to prioritise can support engagement and motivation. While such techniques did not meet the predefined consensus threshold, they may still hold significant value in specific clinical contexts and intervention phases. Despite these considerations, the modified Delphi approach remains a robust method for identifying expert agreement on core MI-CBT components.

### Conclusion

This modified Delphi study established expert consensus regarding the essential components of integrated MI-CBT interventions for lifestyle behaviour change in PA, diet and smoking. The final consensus included eight relational, 16 content, and two process components that provide a structured foundation for integrated MI-CBT interventions. Rather than serving as a prescriptive framework, these components offer guidance for clinicians and researchers seeking to design, deliver, and evaluate MI-CBT interventions. These findings may assist clinicians who integrate behaviour change techniques into their practice, helping them prioritise key strategies. For researchers, this consensus provides a foundation for developing integrated MI-CBT interventions that can be tested against alternative or emerging approaches. However, further validation is needed before these components can be formally adopted as standardised guidelines or fidelity tools. Future research should focus on evaluating the implementation and effectiveness of these core components in real-world settings.

## Supplementary Information

Supplementary Material 1.

## Data Availability

The datasets used and/or analysed during the current study are available from the corresponding author on reasonable request and subject to approval from the governing HREC.

## Abbreviations

CBT: cognitive behavioural therapy
MI-CBT: motivational interviewing and cognitive behavioural therapy
MI: motivational interviewing
PA: physical activity
