# Supplementary material for: Effective components of integrated motivational interviewing and cognitive behavioural therapy for lifestyle behaviour change: a modified Delphi study
Source: Int J Behav Nutr Phys Act. 2025 Nov 3;22:135. doi: 10.1186/s12966-025-01816-6 (PMC12581319; doi:10.1186/s12966-025-01816-6)
Supplement: Supplementary file 1 — Supplementary Material 1. [file 12966_2025_1816_MOESM1_ESM.pdf]

Additional file 1. Delphi Checklist

**Rationale for the choice of the Delphi technique**	Details (where in the manuscript is this item addressed)
1. *Justification.* The choice of the Delphi technique as a method of systematically collating expert consultation and building consensus needs to be well justified. When selecting the method to answer a particular research question, it is important to keep in mind its constructivist nature	Introduction ->Final paragraph.
**Planning and design**	
2. *Planning and process.* The Delphi technique is a flexible method and can be adjusted to the respective research aims and purposes. Any modifications should be justified by a rationale and be applied systematically and rigorously	Methods -> Study design
3. *Definition of consensus.* Unless not reasonable due to the explorative nature of the study, an a priori criterion for consensus should be defined. This includes a clear and transparent guide for action on (a) how to proceed with certain items or topics in the next survey round, (b) the required threshold to terminate the Delphi process and (c) procedures to be followed when consensus is (not) reached after one or more iterations	Methods -> Consensus
**Study conduct**	
4. *Informational input.* All material provided to the expert panel at the outset of the project and throughout the Delphi process should be carefully reviewed and piloted in advance in order to examine the effect on experts' judgements and to prevent bias	Methods -> Data collection
5. *Prevention of bias.* Researchers need to take measures to avoid directly or indirectly influencing the experts' judgements. If one or more members of the research team have a conflict of interest, entrusting an independent researcher with the main coordination of the Delphi study is advisable	Refer to Declarations of conflict of interest. 'The authors declare that there are no known conflicts of interest related to this project that could have influenced this manuscript.
6. *Interpretation and processing of results.* Consensus does not necessarily imply the 'correct' answer or judgement; (non)consensus and stable disagreement provide informative insights and highlight differences in perspectives concerning the topic in question	Methods -> Data analysis
7. *External validation.* It is recommended to have the final draft of the resulting guidance on best practice in palliative care reviewed and approved by an external board or authority before publication and dissemination	Not applicable. All authors have read and approved the manuscript submitted to the journal for publication.
Reporting	
8. *Purpose and rationale.* The purpose of the study should be clearly defined and demonstrate the appropriateness of the use of the Delphi technique as a method to achieve the research aim. A rationale for the choice of the Delphi technique as the most suitable method needs to be provided	Introduction ->Final paragraph.

9. *Expert panel.* Criteria for the selection of experts and transparent information on recruitment of the expert panel, socio-demographic details including information on expertise regarding the topic in question, (non)response and response rates over the ongoing iterations should be reported	Methods -> Participants and recruitment and 'Figure 1: Flow diagram presenting the modified Delphi study process
10. *Description of the methods.* The methods employed need to be comprehensible; this includes information on preparatory steps (How was available evidence on the topic in question synthesised?), piloting of material and survey instruments, design of the survey instrument(s), the number and design of survey rounds, methods of data analysis, processing and synthesis of experts' responses to inform the subsequent survey round and methodological decisions taken by the research team throughout the process	Methods
11. *Procedure.* Flow chart to illustrate the stages of the Delphi process, including a preparatory phase, the actual 'Delphi rounds', interim steps of data processing and analysis, and concluding steps	Figure 1: Flow diagram presenting the modified Delphi study process
12. *Definition and attainment of consensus.* It needs to be comprehensible to the reader how consensus was achieved throughout the process, including strategies to deal with non-consensus	Methods -> Consensus
13. *Results.* Reporting of results for each round separately is highly advisable in order to make the evolving of consensus over the rounds transparent. This includes figures showing the average group response, changes between rounds, as well as any modifications of the survey instrument such as deletion, addition or modification of survey items based on previous rounds	Results -> including Table 1 and 2
14. *Discussion of limitations.* Reporting should include a critical reflection of potential limitations and their impact of the resulting guidance	Discussion -> Paragraph 6
15. *Adequacy of conclusions.* The conclusions should adequately reflect the outcomes of the Delphi study with a view to the scope and applicability of the resulting practice guidance	Conclusion, and Abstract Conclusion
16. *Publication and dissemination.* The resulting guidance on good practice in palliative care should be clearly identifiable from the publication, including recommendations for transfer into practice and implementation. If the publication does not allow for a detailed presentation of either the resulting practice guidance or the methodological features of the applied Delphi technique, or both, reference to a more detailed presentation elsewhere should be made (e.g. availability of the full guideline from the authors or online; publication of a separate paper reporting on methodological details and particularities of the process (e.g. persistent disagreement and controversy on certain issues)). A dissemination plan should include endorsement of the guidance by professional associations and health care authorities to facilitate implementation	Not applicable.

Recommendations for the Conducting and REporting of DElphi Studies (CREDES).

Source: Jünger S, Payne SA, Brine J, Radbruch L, Brearley SG. Guidance on Conducting and REporting DElphi Studies (CREDES) in palliative care: Recommendations based on a methodological systematic review. Palliative Medicine. 2017;31(8):684-706. doi:10.1177/02692163176906

Additional file 2. List of components

Relational Components

Relational components reflects the attitude and interview style of the practitioner that together facilitate the development of an effective client-centred collaborative relationship.

1. Open ended questions
2. Affirmations
3. Reflections
4. Summaries
5. Emphasise autonomy
6. Understating

   Definition: a reflection that diminishes or understates the intensity of the content or emotion expressed by a client.

7. Coming alongside the client

   Definition: a response to persistent sustain talk or discord in which the interviewer adopts and reflects the person's perspective

8. Agreement with a twist

   Definition: A reflection, affirmation, or accord followed by a reframe

9. Overstating

   Definition: a reflection that adds intensity to the content or emotion that was expressed.

10. Offer emotional support
11. Agenda mapping

    Definition: selecting from a menu of possibilities the topic(s) to be discussed in a consultation

12. Review a typical day
13. Ask-offer-ask

    Definition: an information exchange process that begins and ends with exploring the person's own experience to frame whatever information is being provided

14. Shifting attention

    Definition: a way of responding to discord by redirecting attention and discussion to a less contentious topic or perspective

15. Double-sided reflection

Content components

Content components reflects the therapeutic behaviour change techniques used by the practitioner to facilitate behaviour change. Using this Delphi process we wish to reach consensus on the necessary content components of MI-CBT interventions for lifestyle behaviour change

1. Activity monitoring
2. Agreeing on a list of priority problems
3. Behavioural experiments to explore evidence for beliefs and assumptions
4. Develop a Change Plan (CATs) C= Commitment A= Activation T= Taking steps
5. Creating a written agenda that is agreed collaboratively at the start of each session
6. Ensuring that the client understands the model/rationale for MI-CBT
7. Developing a formulation and using it to outline a treatment plan
8. Eliciting feedback to ensure a shared understanding and adapting therapy based on feedback
9. Developing and maintaining a good therapeutic alliance and understanding of the client's perspective
10. Agreeing on a list of priority goals
11. Explore change expectations
12. Exposure techniques
13. Goal attainment scaling
14. Identify strengths
15. Identify past successes
16. Identifying and exploring avoidant behaviour
17. Identifying and challenging key cognitions including negative automatic thoughts
18. Identifying and challenging unhelpful thinking styles (e.g. all or nothing thinking)
19. Identifying and modifying conditional beliefs/underlying assumptions/rules for living
20. Identifying and exploring core beliefs
21. Looking forward and looking back
22. Managing difficult emotions and helping the client to test out the associated beliefs
23. Managing the ending of a course of therapy
24. Methods to prevent relapse
25. Planning and reviewing practice ("homework") assignment
26. Providing the client with written worksheets to gather information, or practice new skills

27. Providing a written summary of what was covered in each session - including goals for the period that follows
28. Reframing
29. Review outcome goal
30. Summarise the plan
31. Undertaking an initial assessment (including translating abstract complaints into concrete and discrete problems)
32. Use of importance and confidence rulers
33. Using questions to help the client make his/her own discoveries (i.e. guided discovery and Socratic questioning)
34. Self-monitoring
    Definition: The practice of tracking and documenting personal experiences, such as thoughts and feelings to gain insight into how these elements are interconnected and how they influence one's behaviour.
35. Activity scheduling
36. Problem solving
    Definition: A systematic process that involves identifying problems, generating potential solutions, evaluating and selecting the best options, and implementing these solutions. The goal is to enhance clients' ability to cope with and manage their problems effectively

Process components

Process components reflects the structure and delivery of the MI-CBT sessions. Using this Delphi process we wish to reach consensus on the necessary process components of MI-CBT interventions for lifestyle behaviour change

1. Ensuring that the therapist receives regular supervision
2. Ensuring that MI-CBT is provided by a therapist who meets recognised standards for MI-CBT training and practice
3. Ensuring that the therapist has been shown to be 'competent' in the delivery of MI-CBT using an established competency checklist
4. Scheduling MI-CBT sessions flexibly according to client need
5. Scheduling each MI-CBT session to last less than 30 minutes
6. Providing at least 5 sessions of MI-CBT
7. Scheduling MI-CBT to take place at least once per week for the majority of the therapy
8. Providing at least 12 sessions of MI-CBT
9. Providing additional support between MI-CBT sessions by telephone check-in, email and/or instant messaging
10. Providing at least 13-20 sessions of MI-CBT
11. Providing MI-CBT face-to-face rather than by telephone or video conference
12. Providing advance reminders of sessions e.g. by text, email or letter
